# A Shaving Proteomic Approach to Unveil Surface Proteins Modulation of Multi-Drug Resistant *Pseudomonas aeruginosa* Strains Isolated From Cystic Fibrosis Patients

**DOI:** 10.3389/fmed.2022.818669

**Published:** 2022-03-09

**Authors:** Anna Lisa Montemari, Valeria Marzano, Nour Essa, Stefano Levi Mortera, Martina Rossitto, Simone Gardini, Laura Selan, Gianluca Vrenna, Andrea Onetti Muda, Lorenza Putignani, Ersilia Vita Fiscarelli

**Affiliations:** ^1^Department of Diagnostics and Laboratory Medicine, Unit of Microbiology and Diagnostic Immunology, Unit of Cystic Fibrosis Diagnostics, Bambino Gesù Children's Hospital IRCCS, Rome, Italy; ^2^Multimodal Laboratory Medicine Research Area, Unit of Human Microbiome, Bambino Gesù Children's Hospital IRCCS, Rome, Italy; ^3^GenomeUp, Rome, Italy; ^4^Department of Public Health and Infectious Diseases, Sapienza University, Rome, Italy; ^5^Department of Diagnostics and Laboratory Medicine, Bambino Gesù Children's Hospital IRCCS, Rome, Italy; ^6^Department of Diagnostics and Laboratory Medicine, Unit of Microbiology and Diagnostic Immunology, Unit of Microbiomics, and Multimodal Laboratory Medicine Research Area, Unit of Human Microbiome, Bambino Gesù Children's Hospital IRCCS, Rome, Italy

**Keywords:** cystic fibrosis, *Pseudomonas aeruginosa*, antibiotic resistance, shaving proteomics, long-term colonization

## Abstract

Cystic fibrosis (CF) is the most common rare disease caused by a mutation of the *CF transmembrane conductance regulator* gene encoding a channel protein of the apical membrane of epithelial cells leading to alteration of Na^+^ and K^+^ transport, hence inducing accumulation of dense and sticky mucus and promoting recurrent airway infections. The most detected bacterium in CF patients is *Pseudomonas aeruginosa* (PA) which causes chronic colonization, requiring stringent antibiotic therapies that, in turn induces multi-drug resistance. Despite eradication attempts at the first infection, the bacterium is able to utilize several adaptation mechanisms to survive in hostile environments such as the CF lung. Its adaptive machinery includes modulation of surface molecules such as efflux pumps, flagellum, pili and other virulence factors. In the present study we compared surface protein expression of PA multi- and pan-drug resistant strains to wild-type antibiotic-sensitive strains, isolated from the airways of CF patients with chronic colonization and recent infection, respectively. After shaving with trypsin, microbial peptides were analyzed by tandem-mass spectrometry on a high-resolution platform that allowed the identification of 174 differentially modulated proteins localized in the region from extracellular space to cytoplasmic membrane. Biofilm assay was performed to characterize all 26 PA strains in term of biofilm production. Among the differentially expressed proteins, 17 were associated to the virulome (e.g., Tse2, Tse5, Tsi1, PilF, FliY, B-type flagellin, FliM, PyoS5), six to the resistome (e.g., OprJ, LptD) and five to the biofilm reservoir (e.g., AlgF, PlsD). The biofilm assay characterized chronic antibiotic-resistant isolates as weaker biofilm producers than wild-type strains. Our results suggest the loss of PA early virulence factors (e.g., pili and flagella) and later expression of virulence traits (e.g., secretion systems proteins) as an indicator of PA adaptation and persistence in the CF lung environment. To our knowledge, this is the first study that, applying a shaving proteomic approach, describes adaptation processes of a large collection of PA clinical strains isolated from CF patients in early and chronic infection phases.

## Introduction

Cystic fibrosis (CF) is a rare disease caused by mutations of the *CF transmembrane conductance regulator* gene (CFTR) which encodes for a channel protein regulating Na^+^ and K^+^ transportation in and out of the epithelial cells (1). Several genetic mutations of the CFTR gene are implicated in the pathogenesis of the disease giving rise, along with other factors, to a multitude of ailment phenotypes (2). Regardless of the type of CFTR dysregulations, the effect results in systemic disease, with the major issues represented by the generation of dense and sticky mucus deeply affecting lung pathophysiology (3) and colonization of both Gram-positive and Gram-negative bacteria, as well as fungal colonization (4, 5). Therefore, CF is characterized by recurrent pulmonary exacerbations, a decrease of the lung function and increased morbidity and mortality, requiring more stringent antimicrobial therapies which, in turn, trigger bacteria antibiotic resistance (AR) (6–8). The resulting antibiotic resistance (AR) is a crucial issue. In 2017 the World Health Organization (WHO) listed critical microorganisms for which it is imperative to develop new drugs, that included the multi-drug resistant (MDR) ESKAPE (i.e., *Enterococcus faecium, Staphylococcus aureus, Klebsiella pneumoniae, Acinetobacter baumannii, Pseudomonas aeruginosa*, and *Enterobacter* species) (9).

The Gram-negative *P. aeruginosa* (PA) is the most isolated bacterium in CF in children and teens and its prevalence increases in adulthood with up to 70% of overall grownup CF subjects (5). Moreover, the 13.2% of all PA colonized patients have multi-drug resistant PA (Cystic Fibrosis Foundation Patient Registry, Annual Data Report 2020).

Antibiotic resistance is the result of PA ability to acquire resistance through genes, plasmids, membrane permeability, and/or chromosomal mutations (10), and is part of the so-called “adaptive radiation”. This phenomenon includes loss of motility and virulence factors associated with early infection; modifications of adherence and biofilm; the appearance of late colonization virulence factors; and changes in growth rates and porins' expression (1, 11, 12). This high plasticity makes the pathogen extremely flexible and capable of surviving in the hostile CF pulmonary environment, which is characterized by low oxygen, viscous mucus, high competition with other bacteria, fluctuating pH, the presence of host innate immunity and antibiotic molecules (13).

Adaptation mechanisms reflect re-arrangements of the bacterial proteins on the surface of the microbe, suggesting that antibiotic resistant PA isolates from long-term colonization may express a different surface protein layout compared to antibiotic sensitive wild type PA isolates from recent infection. Mass spectrometry-driven proteomics is a powerful and valuable approach as it provides the chance to simultaneously identify and quantify several proteins (14). Different proteomic procedures to characterize surface proteins (the so-called “surfaceome”) may be employed including extraction and enrichment methods based on subcellular fractionation (15), cell surface coating or biotinylation (16) and the “shaving” of live bacterial cells using proteases (17, 18). The advantage of the “shaving” approach resides in directly targeting live bacterial cells with no need for extensive biochemical purifications in a fast and reliable way. The methodology was employed to ascertain potential vaccine candidates (19–22) or study biofilm formation (23) and was first described for Gram-positive bacteria (19). Afterwards, a plethora of scientific works reported shaving proteomics applied to prokaryotes and eukaryotes, irrespective of protease variations, quantity, and incubation time. The procedure is still mainly utilized on Gram-positive bacteria (19, 20, 22–35), but also suitable for Gram-negative bacteria (21, 33, 36–38), despite intrinsic limitation of the method associated with the retention of cytoplasmic proteins (28–30). However, cell lysis may not be the only explanation for the presence of cytoplasmic proteins in the shaved fraction. Indeed, there are proteins predicted as intracellular but exported by unknown secretion pathways or even delivered to extracellular space (i.e., the so-called “Moonlighting” proteins) (17). The importance of these proteins, identified as both intra- and extra-cellular, may come from a potential crucial role as virulence factors (39) or enzymes involved in immunological escape (40).

So far, the only paper utilizing a shaving strategy in PA attempted to expand the knowledge of proteins interacting with the environment (37).

In the present study the bacterial shaving proteomics was exploited to study surface proteins of PA in clinical strains isolated from CF patients characterized by multi- and pan-drug antibiotic resistance profiles compared to antibiotic sensitive strains. These findings may contribute to the comprehensive landscape description of PA surface proteins related to antibiotics resistance and its resulting virulence profile. The characterization of modulated proteins may be the key to decode the functional activity of PA infection and antibiotic resistance in CF, thus having a valuable impact in unveiling new virulence markers and therapeutic targets.

## Materials and Methods

### *Pseudomonas aeruginosa* Strains and Culture Conditions

Nine multi-drug and seven pan-drug resistant (MDR and PDR, respectively) non-mucoid strains of *P. aeruginosa* isolated from the airways of CF patients with chronic colonization (4–15 years, except patient # 1), nine wild-type (WT) antibiotic-sensitive strains isolated from the airways of CF patients with recent infection (<12 months), and a PAO1 reference strain were analyzed (WT_10) (Table 1; Supplementary Table 1). According to The European Committee on Antimicrobial Susceptibility Testing (http://www.eucast.org), WT strains are defined as sensitive to all antimicrobials, while MDR strains are resistant to at least one agent in three or more antimicrobial categories and PDR strains are resistant to all antibiotics in all classes.

**Table 1:** List of studied *P. aeruginosa* strains and their antibiotic susceptibility profiles.

**Group**	**Strain**	**Fluoroquinolones**		**Penicillins**	**Monobactams**	**Cephalosporins**		**Aminoglycosides**		**Carbapenems**		**Polimixine**	**Combination**	
		**Ciprofloxacin**	**Levofloxacin**	**Piperacillin-tazobactam**	**Aztreonam**	**Ceftazidime**	**Cefepime**	**Amikacin**	**Tobramycin**	**Imipenem**	**Meropenem**	**Colistin**	**Ceftazidime-avibactam**	**Ceftolozane-tazobactam**
MDR	MDR_01	R	R	R	R	R	R	R	R	R	R	S	R	R
	MDR_02	I	R	R	R	R	R	R	R	R	R	S	R	R
	MDR_03	I	R	R	R	R	R	R	R	R	I	X	S	R
	MDR_04	I	R	R	R	R	R	R	R	R	I	S	S	S
	MDR_05	R	R	R	R	R	R	R	R	R	R	S	S	S
	MDR_06	R	R	S	S	S	S	R	R	R	R	S	X	X
	MDR_07	R	R	R	R	R	R	R	S	R	R	S	S	S
	MDR_08	R	R	R	R	R	R	S	S	R	I	S	S	R
	MDR_09	R	R	I	R	S	R	R	R	I	R	S	S	S
PDR	PDR_01	R	R	R	R	R	R	R	R	R	R	R	R	R
	PDR_02	R	R	R	I	R	R	R	R	R	R	R	R	R
	PDR_03	R	R	R	R	R	R	R	R	R	R	R	X	X
	PDR_04	R	R	R	R	R	R	R	R	R	R	R	R	R
	PDR_05	R	R	R	R	R	R	R	R	R	R	R	R	R
	PDR_06	R	R	R	R	R	R	R	R	R	R	R	R	R
	PDR_07	R	R	R	R	R	R	R	R	R	R	R	R	R
WT	WT_01	I	R	I	I	I	I	S	S	I	S	S	X	X
	WT_02	I	I	I	I	I	I	S	S	I	S	S	X	X
	WT_03	I	I	I	X	I	I	S	S	I	S	S	S	S
	WT_04	I	I	I	X	I	I	S	S	I	S	X	S	S
	WT_05	I	I	S	X	I	I	S	I	X	S	X	X	X
	WT_06	I	I	I	X	I	I	S	S	I	S	S	S	S
	WT_07	I	I	I	I	I	I	S	S	I	S	S	X	X
	WT_08	I	I	I	X	I	I	S	S	I	S	S	S	S
	WT_09	I	I	I	X	I	I	S	S	I	S	S	S	S
	WT_10	S	S	S	S	S	S	S	S	S	S	S	S	S

*Antibiotic susceptibility was determined according to The European Committee on Antimicrobial Susceptibility Testing (version 11.0, 2021, http://www.eucast.org); S, Susceptible, standard dosing regimen; I, Susceptible, increased exposure; R, Resistant; X, not tested*.

After isolation, all strains were kept at −80°C in Cryobank tubes (Mast Group Ltd., Bootle, Merseyside, United Kingdom) until they were ready for processing. Bacteria were plated on Columbia agar with 5% sheep blood (Becton Dickinson GmbH, Heidelberg, Germany), and then grown in Brain-Heart infusion broth (BHI, bioMérieux Italia S.p.A., Bagno a Ripoli, Firenze, Italy) at 37°C until reaching the mid-exponential phase (OD_600_ = 0.4).

### Bacterial Shaving

Ten mL of bacterial growth was centrifuged 15 min at 4,000 *g*, 4°C; the pellet was suspended in 10 mL of ice-cold phosphate buffer (DPBS; KCl 200 mg/L, KH_2_PO_4_ 200 mg/L, NaCl 8,000 mg/L, Na_2_HPO_4_ 1150 mg/L) with 20% sucrose, washed two times and resuspended in 1 mL of the same buffer. Shaving was performed by adding 2.5 μg/mL of sequencing-grade trypsin (Promega, Milan, Italy), keeping the solution at 37°C, 5 min, in agitation (60 rpm) on a Forma Orbital Shaker (Thermo Fisher Scientific, Waltham, MA, USA). After centrifugation to remove cells, the supernatant was filtered through a 0.2 μm filter. Fifty percent (50%) of the resulting supernatant was treated with 1 mM Dithiothreitol and 1 mM Iodacetammide, then digested by adding 0.5 μg of fresh trypsin overnight at 37°C. The reaction was stopped by adding a final concentration of 0.1% Trifluoroacetic Acid (TFA) and the sample was desalted on Pierce C18 Spin Columns (Thermo Fisher Scientific). Peptides were speedvac-dried, and resuspended in a water solution with 2% Acetonitrile (ACN) and 0.1% Formic Acid (FA). The total peptide content was determined by a NanoDrop 2000 (Thermo Fisher Scientific), with a standard curve of MassPrep *Escherichia coli* digestion (Waters, Milford, MA, USA).

### NanoLiquid Chromatography-ElectroSpray Ionization-Tandem Mass Spectrometry (nLC-ESI-MS7MS) Analysis

Separation by reverse-phase chromatography, identification and quantification of shaved proteins were carried out on an UltiMate3000 RSLCnano System coupled with an Orbitrap Fusion Tribrid mass spectrometer (Thermo Fisher Scientific) equipped with a nanoESI source (EASY-Spray NG), as already described elsewhere (41) with minor modifications.

Peptides (1.71 μg) were loaded onto a micro-column (C18 PepMap100, Thermo Fisher Scientific) for trapping and desalting. Separation was then performed by a 60 min linear gradient starting from 95% solution A (0.1% FA in water) to 25% solution B (99.9% ACN, 0.1%FA) on an EASY-Spray PepMap RSLC C18 column (2 μm particle size, 100 Å pore size, 75 μm i.d. × 50 cm length, Thermo Fisher Scientific), at a flow rate of 250 nL/min and a temperature of 35°C. Precursor MS survey scans were detected within the range of 375–1,500 *m/z* by the Orbitrap operating in positive ionization mode, at resolving power of 120 K (at 200 *m/z*), setting the automatic gain control target at 4.0 × 10^5^ ions and the maximum injection time at 50 ms. Data dependent MS/MS analysis was performed in top speed mode with a 3 s cycle-time, alternating MS and MS/MS experiments, during which the most abundant multiple-charged (2^+^ – 7^+^) precursor ions, with a signal intensity threshold of 5 × 10^3^, were subjected to high-energy collisional dissociation using 30% normalized collision energy. Ion fragments were acquired by the Ion Trap detector applying of 2.0 × 10^3^ ions as the automatic gain control target and 300 ms as the maximum injection time.

### Protein Identification

Mass spectrometry raw data processing was obtained by Proteome Discoverer software (PD, version 2.4, Thermo Fisher Scientific) interrogating the *P. aeruginosa* UniProtKB reference proteome database (UP000002438, release: 2020_04, 5,564 proteins) and 39 common contaminant sequence entries. The search engine (Sequest HT) parameters included trypsin as the proteolytic enzyme, with a maximum of 2 missed cleavages per peptide allowed, oxidation of methionine as variable modification, carbamidomethylation of cysteine as static modification, precursor mass tolerance threshold ≤ 10 ppm, and maximum fragment mass tolerance = 0.6 Da. For protein identifications, the false-discovery rate (FDR) cut-off was set at 0.01, based on Percolator algorithm and on a decoy database. Contaminant proteins were filtered out and only proteins with at least two identified peptides were considered.

MS dataset and search engine result files are available via MassIVE and ProteomeXchange public repositories with identifiers MSV000088468 and PXD030040 (http://proteomecentral.proteomexchange.org/cgi/GetDataset?ID=PXD030040), respectively.

### Data Analysis and Relative Quantification

Label-free quantitation (LFQ) (42) was performed by PD applying the following parameters: precursor abundance quantification based on intensity, normalization mode on total peptide amount, and protein abundance calculation performed by summing sample abundances of the connected peptide groups.

Taking as input the complete protein normalized intensities matrix obtained from PD software, a filter was applied for each group removing all the proteins that were not present in at least 75% of the samples (MDR, PDR, and WT). All missing values were filled with zeros, and two unsupervised (Bray-Curtis beta diversity and Principal Component Analysis, PCA) analyses were explored. The aforementioned analyses were plotted by showing the first two components; samples were colored by group and the covariance confidence ellipses (σ = 1.4) were shown. Permutational multivariate analysis of variance (PERMANOVA, 9,999 permutations) was performed in order to test the association between covariates and beta diversity measures. To perform the pre-processing and the statistical analyses, *ad-hoc* Python 3.7 scripts were used (main packages: pandas, numpy, scipy, vegan, and scikit-learn).

Hierarchical clustering analysis was performed by the PD software on the complete data matrix of normalized protein abundances applying a *z*-score transformation and computing the distance function by Pearson product-moment correlation and the linkage function by the greatest distance.

The PD protein ratio calculation between paired sample groups was based on *t*-test ratio (median of all possible pairwise peptide ratios calculated between replicates of all connected peptides), presence of each feature in at least 75% of samples in one group, and adjusted *p*-value < 0.05, using Benjamini-Hochberg correction for the FDR.

To move from relative quantification of identified proteins to functional analysis, Kyoto Encyclopedia of Genes and Genomes (KEGG) pathway (https://www.genome.jp/kegg/) and literature searches were utilized.

### Prediction of Protein Subcellular Localization

Subcellular localization of the identified proteins was retrieved merging information from several web-based tools driven by diverse features-based algorithms, such as the presence of classical and non-classical signal peptides, the amino acid composition and putative transmembrane helices. The analyses were performed by PSORTb-3.0 (http://www.psort.org/psortb/), SignalP-5.0 (http://www.cbs.dtu.dk/services/SignalP/), TargetP-2.0 (http://www.cbs.dtu.dk/services/TargetP/), LipoP 1.0 (http://www.cbs.dtu.dk/services/LipoP/), CELLO v.2.5 (http://cello.life.nctu.edu.tw/), SecretomeP-2.0 (http://www.cbs.dtu.dk/services/SecretomeP/), TOPCONS (https://topcons.cbr.su.se/), TMHMM - 2.0 (http://www.cbs.dtu.dk/services/TMHMM/), DeepTMHMM (https://dtu.biolib.com/DeepTMHMM), UniProtKB database (https://www.uniprot.org/) and PD ProteinCenter annotation (http://webservice.proteincenter.thermofisher.com/ProXweb/).

### Biofilm Production Assay

The quantification of biofilm production was based on the microtiter plate (MTP) biofilm assay (43). Briefly, the wells of a sterile 96-well flat-bottomed polystyrene plate were filled with 100 μL of BHI. One/100 dilution of overnight bacterial cultures was added into each well (OD_600__nm_ ≈ 0.5). The plates were incubated aerobically for 18 h at 37°C and then planktonic cells were gently removed. Each well was washed three times with double-distilled water, patted dry with a piece of paper towel in an inverted position and stained with 100 μL of 0.1% crystal violet. After 15 min, the MTP was rinsed twice with double-distilled water, and thoroughly dried. The dye bound to adherent cells was solubilized with 20% (v/v) glacial acetic acid and 80% (v/v) ethanol. After 30 min of incubation at room temperature, OD_590nm_ was measured to quantify the total biomass of biofilm formed in each well. Each data point is composed of four independent experiments, each performed in at least 6-replicates. Bacteria with any degree of biofilm production (“weak,” “moderate,” or “strong”) were considered as producers (44).

## Results

### *Pseudomonas aeruginosa* Surface Proteome

Twenty-six *P. aeruginosa* strains were included in the study of which nine MDR and seven PDR clinical isolates from CF patients with chronic colonization, nine antibiotic sensitive WT clinical isolates from CF patients with recent first infection, and an antibiotic sensitive reference strain PAO1 (Table 1).

**Figure 1:**
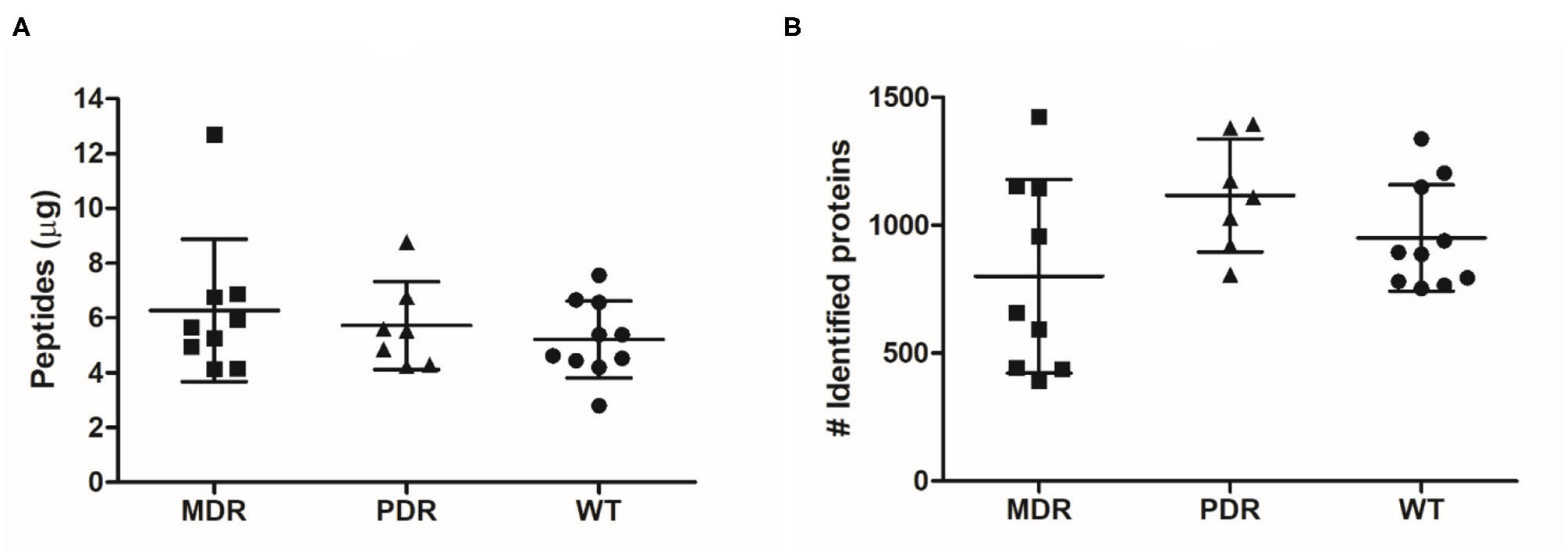
Graph distribution of purified peptides and identified proteins. **(A)** After the shaving procedure, a mean of 6.27 μg (±2.60 standard deviation), 5.72 μg (±1.60) and 5.21 μg (±1.40) of peptides in the MDR, PDR, and WT samples, respectively, were purified. **(B)** By nLC–ESI–MS/MS, we identified a mean of 801 (±379), 1,117 (±221), and 950 (±208) proteins were identified in the MDR, PDR, and WT groups, respectively.

Through shaving proteomics, we purified a mean of 6.27 μg (±2.60 standard deviation, s.d.) of peptides in the MDR group, 5.72 μg (±1.60 s.d.) in PDR samples, and 5.21 μg (±1.40 s.d.) in the WT group (Figure 1A). By nLC–ESI–MS/MS, applying ID filters and removing contaminants, 2,206 (mean of 801 ± 379 s.d.), 2,238 (1,117 ± 221 s.d.), and 2,170 (950 ± 208 s.d.) total diverse proteins were identified in the MDR, PDR and WT groups, respectively (Figure 1B; Supplementary Table 2). When performing proteomic mass spectrometry-based experiments, contamination events are not completely avoidable and in our experiments are quite low, amounting to a mean of 0.42 ± 0.28% of proteins respective to the total identified proteins for each strain. The contaminants derive from keratins from human skin/hair, clothes made of sheep wool, or lab dust; autolytic digestion of the used protease; and rubber from labware equipment (45, 46). The quantity of purified peptides allowed an optimal nLC-MS/MS analysis and numbers of identified proteins were consistent with other published papers related to shaving proteomics of bacteria (Supplementary Table 3).

### Comparative Analysis of Surface Protein Extracts From Antibiotic-Resistant and Sensitive *Pseudomonas aeruginosa* Strains

LFQ analysis was performed by PD software quantifying 2,370 different proteins across all samples (Supplementary Table 4), covering 43% of the theoretical proteome. This percentage was higher than expected, considering predicted localization results based on the PAO1 complete reference genome (Refseq Accession NC_002516.2), available at PSORTb website, and theoretically mapping 31% of proteins spanning the region from extracellular space to the cytoplasmic membrane. However, this occurrence may be explained by the presence in the shaved fraction of both cytoplasmic proteins derived from cell lysis and moonlighting proteins.

**Figure 2:**
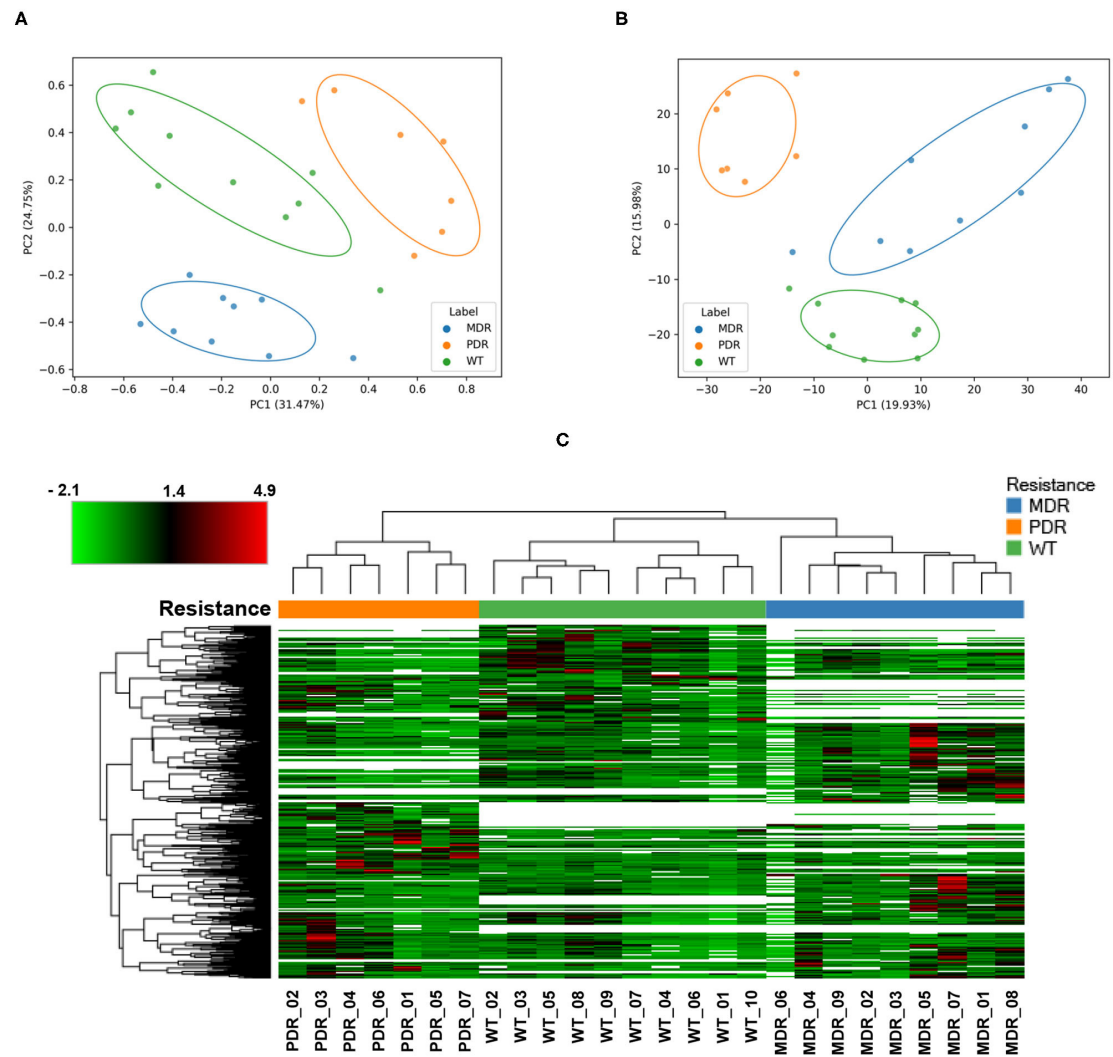
Analyses of quantified proteins. **(A)** The dissimilarity between samples' groups by the unsupervised Bray-Curtis beta diversity analysis was measured according to their proteins' content. MDR, PDR and WT groups showed a statistically significant (*p*-value ≤ 0.001) differences assessed by PERMANOVA test (PC1 vs. PC2; PC1: 31.47%; PC2: 20.75%). **(B)** Unsupervised Principal Component Analysis displayed a good separation amongst groups (PC1 vs. PC2; PC1: 19.93%; PC2: 15.98%). **(C)** Color-coded hierarchical cluster analysis visualized by the heat map based on the normalized protein abundances and applying a z-score transformation. The dendrogram above the heat map, representing the distance between samples, demonstrated good similarity among samples of each strain groups. The top left heat map color legend displays the range of the scaled protein abundance values, ranging from −2.1 to +4.9 and a mean of +1.4. Blue, orange and green color of **(A–C)** labels/resistance profiles correspond to MDR, PDR and WT *P. aeruginosa* strains, respectively.

In order to have an overview of sample classification into subgroups, according to their proteins content, we measured the dissimilarity between samples using beta diversity analysis (Figure 2A). Sample distribution showed a dissimilarity corresponding to the three groups MDR, PDR, and WT (PC1 vs PC2; PC1: 31.47%; PC2: 20.75%). The groups showed statistically significant (*p*-value ≤ 0.001) differences assessed by the PERMANOVA test.

Also PCA analysis displayed a good separation amongst groups (PC1 vs. PC2; PC1: 19.93%; PC2: 15.98%) outlining a segregation between antibiotic-susceptible and -resistant strains along the PC2 axis; the largest intra-group variation was observed in the MDR group (Figure 2B).

Moreover, cluster analysis, visualized by a heat map, showed the bacterial strain clustering into three groups based on similarities (Figure 2C).

**Figure 3:**
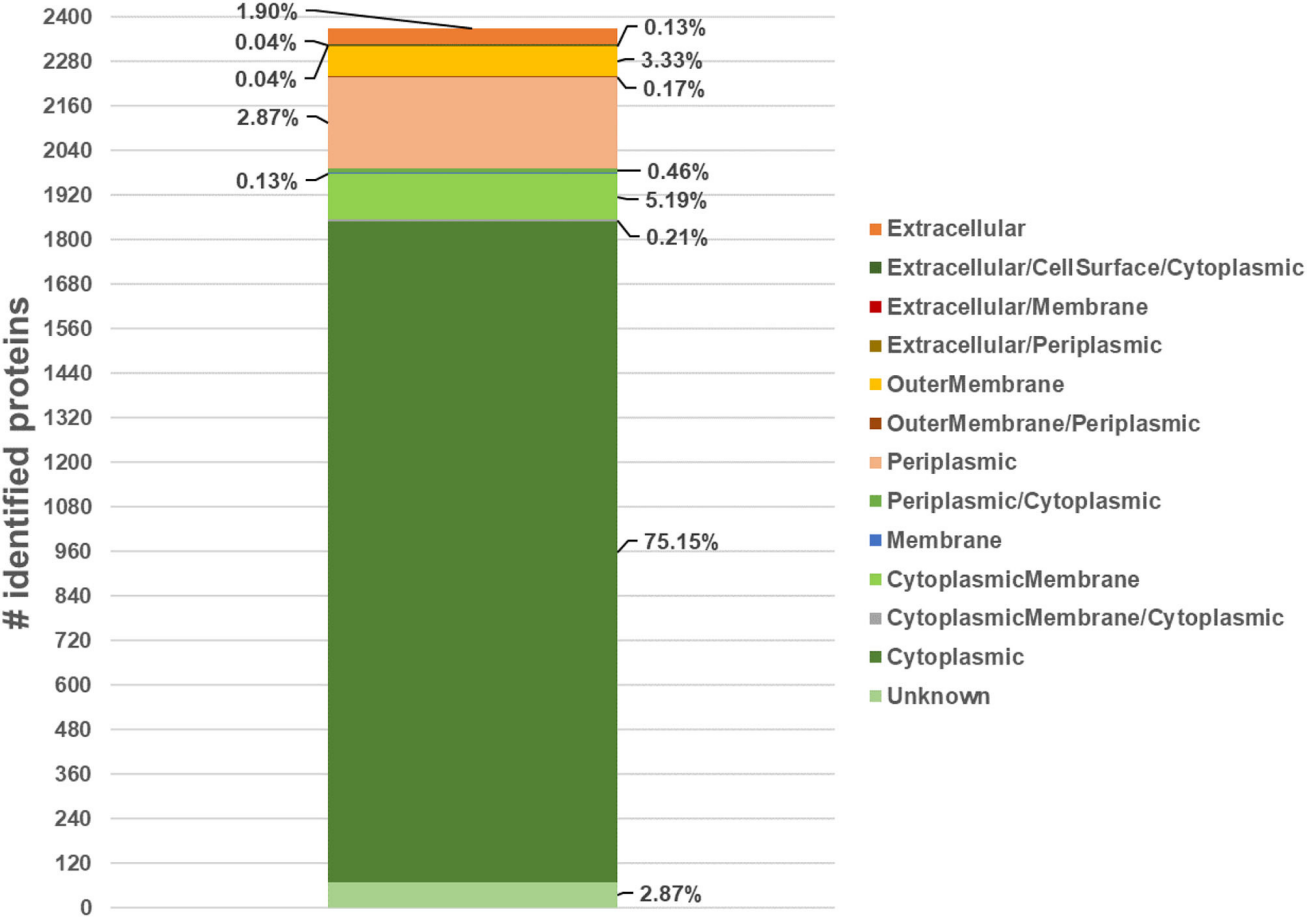
Number of quantified proteins and their subcellular localization, expressed as percentage respect to the total number of quantified proteins, by merging data from several web-based applications (PSORTb-3.0, SignalP-5.0, TargetP-2.0, LipoP 1.0 CELLO v.2.5, SecretomeP-2.0, TOPCONS, TMHMM-2.0, DeepTMHMM, UniProtKB database and Proteome Discoverer ProteinCenter annotation).

The subcellular localization of the 2,370 quantified proteins mapped 22% of them in the region from extracellular space to the cytoplasmic membrane (Figure 3; Supplementary Table 4). Seventy-five percent of proteins were localized into the cytoplasmic compartment in accordance with previous published papers regardless of different experimental conditions (Supplementary Table 3).

In order to explore the expression of proteins likely involved in antibiotic susceptibility, we performed a differential analysis between MDR and WT, and PDR vs. WT groups based on *t*-test and a fold change of at least ± 2.

**Figure 4:**
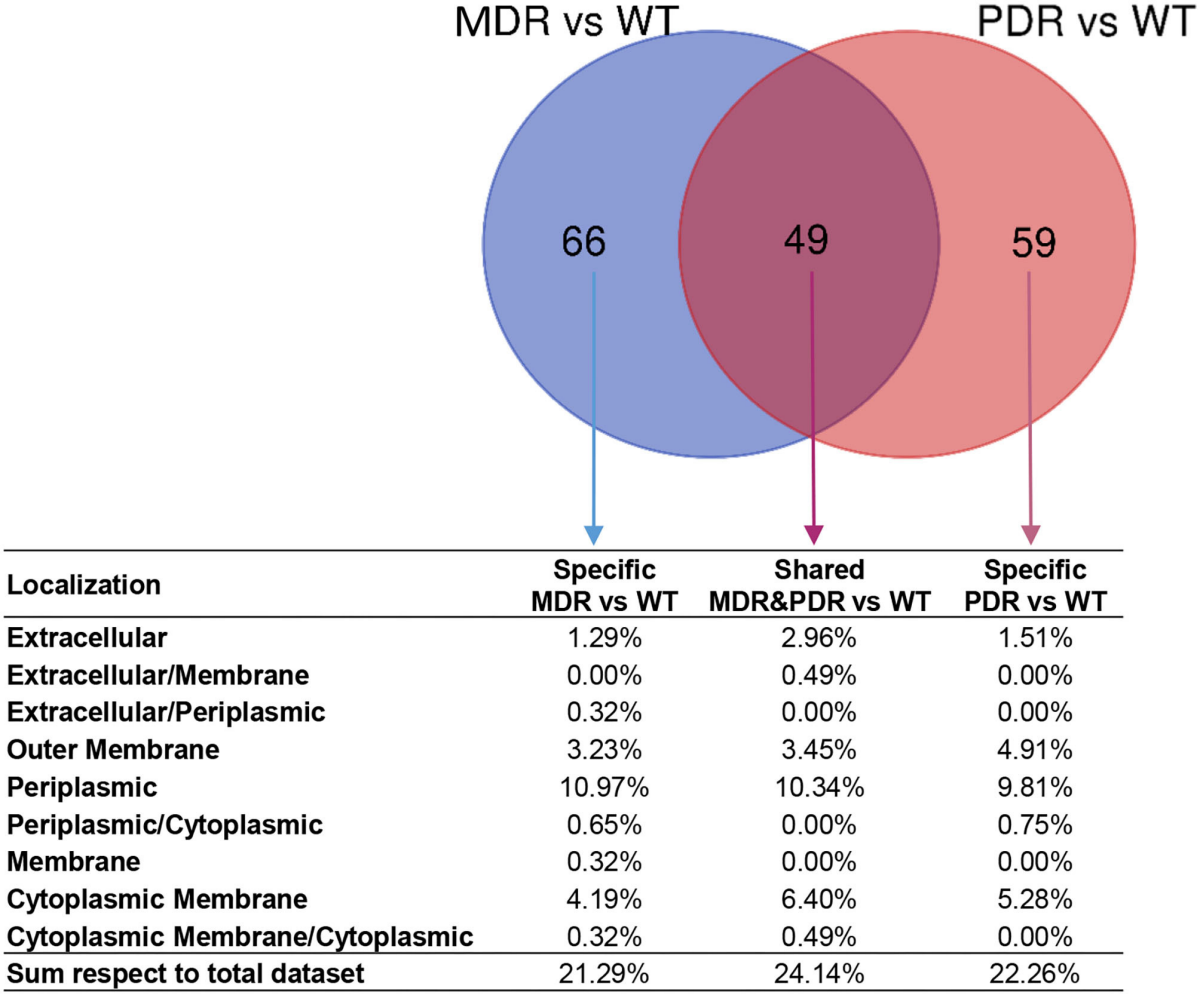
Venn diagram showing common and specific non-cytosolic (extracellular space, cell surface, outer membrane, periplasmic region, and cytoplasmic membrane) proteins in MDR vs. WT (119 total proteins) and PDR vs. WT (108 total proteins) comparisons based on label-free quantification. Percentages of the distribution among the different subcellular regions, respect the total quantified proteins, are reported below each subset of the diagram.

The LFQ analysis revealed that MDR strains showed significantly different levels of 513 proteins compared to WT; the second comparison (PDR vs. WT) led us to identify 468 differentially expressed proteins (Supplementary Data Sheet 1). In detail, 115 and 108 proteins of the MDR and PDR groups, respectively, belonged to extracellular space, cell surface, outer membrane, periplasmic region, and the cytoplasmic membrane. Among these proteins, 49 were present in both comparisons (Figure 4; Table 2).

**Table 2:** List of differential *P. aeruginosa* identified MDR and PDR proteins vs. WT mapped in the region between the extracellular space and the cytoplasmic membrane.

**Accession**	**Gene symbol**	**Description**	**Localization**	**Abundance ratio: (MDR)/(WT)**	**Abundance ratio: (PDR)/(WT)**	**Selected KEGG pathway**
Q9HZQ8	lap	Aminopeptidase	Extracellular	0.01	0.01	
P72151	fliC	B-type flagellin		0.01		pae02040 Flagellar assembly
Q9I589	cbpD	Chitin-binding protein CbpD		0.434		
G3XDA1	exoS	Exoenzyme S			6.325	
Q9I788	exoT	Exoenzyme T		4.584		
P11439	eta	Exotoxin A		0.01	0.01	
Q9HWK6	prpL	Lysyl endopeptidase		0.01	0.01	
Q9HXE0	PA3 866	Pyocin protein			0.01	
Q9I0E0	tse2	Toxin Tse2		100	100	
Q9HVI2	PA4607	Uncharacterized protein			100	
Q9I1B2	PA2 367	Uncharacterized protein (Hcp1 family type VI secretion system effector)		0.01	0.01	pae03070 Bacterial secretion; pae02025 Biofilm formation
Q9I2J0	PA1914	Uncharacterized protein		0.01	0.01	
Q9I3W1	PA1383	Uncharacterized protein		100		
Q9I6D4	PA0360	Uncharacterized protein			100	
Q9I4Y4	pyoS5	Pyocin S5	Extracellular/membrane	100	100	
G3XDA8	pstS	Phosphate-binding protein PstS	Extracellular/periplasmic	0.01		
Q9I2V8	PA1784	Alginate_lyase2 domain-containing protein	OuterMembrane	0.01	0.01	
Q9HVT2	PA4489	Alpha-2-macroglobulin homolog		100		
Q9HU38	PA5146	AsmA domain-containing protein			0.117	
Q9I787	PA0045	Curli production assembly/transport component CsgG			0.01	
O33407	estA	Esterase EstA		100		
P42512	fptA	Fe(3+)-pyochelin receptor			100	
Q9HW32	icmP	Insulin-cleaving metalloproteinase outer membrane protein			100	
Q9I5U2	lptD	LPS-assembly protein LptD		100		
Q9HUX3	cntO	Metal-pseudopaline receptor CntO			100	
Q51397	oprJ	Outer membrane protein OprJ		100	100	
Q51487	oprM	Outer membrane protein OprM			0.01	pae01501 β-Lactam resistance
Q9I4Z4	pal	Peptidoglycan-associated lipoprotein			100	
G3XD11	oprH	PhoP/Q and low Mg2+ inducible outer membrane protein H1			0.01	
Q9HVG7	PA4624	POTRA domain-containing protein		100	100	
Q9I119	PA2463	POTRA domain-containing protein		100		
Q9I5S5	PA0641	Probable bacteriophage protein		0.01		
P30417	fkl	Probable FKBP-type 25 kDa peptidyl-prolyl cis-trans isomerase			100	
Q9HUJ1	PA4974	Probable outer membrane protein		0.01		pae03070 Bacterial secretion; pae01501 β-Lactam resistance
Q9HZU7	PA2900	Probable outer membrane protein		100	100	
Q9I456	PA1288	Probable outer membrane protein		0.01		
G3XD89	oprC	Putative copper transport outer membrane porin OprC		0.01	0.01	
Q9I0K5	PA2633	Secretin_N domain-containing protein		0.01		
Q9I0F4	tse5	Toxin protein Tse5		100	100	
Q9I324	popB	Translocator protein PopB			0.01	
Q9HXJ2	pilF	Type IV pilus assembly protein PilF		0.01	0.01	
G3XD83	PA0625	Uncharacterized protein			0.01	
Q9HV64	PA4735	Uncharacterized protein		0.01		
Q9HZJ1	PA3015	Uncharacterized protein			100	
Q9I124	PA2458	Uncharacterized protein		0.01		
Q9I2D5	PA1969	Uncharacterized protein			0.01	
Q06062	algF	Alginate biosynthesis protein AlgF	Periplasmic	0.01		
Q06749	algL	Alginate lyase			100	
G3XD47	aotJ	Arginine/ornithine binding protein AotJ		0.344		
Q9I2F8	rbsB	Binding protein component of ABC ribose transporter			100	pae02030 Bacterial chemotaxis
Q9HV60	PA4739	BON domain-containing protein			0.134	
Q59635	katB	Catalase		0.01		
Q9I6M0	PA0270	Cupin_2 domain-containing protein		0.01		
P00099	nirM	Cytochrome c-551		0.01	0.026	
P14532	ccpA	Cytochrome c551 peroxidase			0.01	
P72161	pbpG	D-alanyl-D-alanine endopeptidase		0.01	0.01	
Q9I4H1	PA1166	DLH domain-containing protein		0.397		
Q9HU61	PA5123	DUF4124 domain-containing protein		100	100	
Q9I206	PA2109	DUF4142 domain-containing protein		100	100	
Q9I406	ggt	Glutathione hydrolase proenzyme		0.01		
Q9I4M3	PA1112	GSDH domain-containing protein		6.698	0.01	
Q9I2Q0	tsi1	Immune protein Ts *i1*		4.361	6.593	
Q9I6H7	PA0314	L-cysteine transporter of ABC system FliY			0.01	
Q9I2N2	modA	Molybdate-binding periplasmic protein ModA		0.01	0.01	pae02040 Flagellar assembly
Q9HYL2	nosZ	Nitrous-oxide reductase		0.01		
Q9HTI6	PA5378	OpuAC domain-containing protein		0.01		
Q9HU80	PA5103	OpuAC domain-containing protein		0.01		
Q9HUT7	osmE	Osmotically inducible lipoprotein OsmE		0.01	0.01	
Q9HTT0	PA5270	PBPb domain-containing protein		0.01		
Q9I0N8	PA2599	PBPb domain-containing protein			0.01	
Q9I311	bglX	Periplasmic beta-glucosidase		100	100	
Q9I4G4	napB	Periplasmic nitrate reductase, electron transfer subunit		0.01		
P40695	plcR	Phospholipase C accessory protein PlcR		100		
Q9HTR2	pchP	Phosphorylcholine phosphatase		0.01	0.01	
Q9HY16	potD	Polyamine transport protein PotD		0.01	0.01	
Q9HXE1	PA3865	Probable amino acid binding protein		0.01		
Q9HZ48	PA3190	Probable binding protein component of ABC sugar transporter		4.538		
Q9HU87	PA5096	Probable binding protein component of ABC transporter			100	
Q9HUA7	PA5076	Probable binding protein component of ABC transporter		0.01		
Q9HVS5	PA4496	Probable binding protein component of ABC transporter		0.396		pae02030 Bacterial chemotaxis
Q9HWI6	PA4195	Probable binding protein component of ABC transporter		0.01		
Q9I2T4	PA1810	Probable binding protein component of ABC transporter		0.01		
Q9I5T6	PA0602	Probable binding protein component of ABC transporter		0.01		
Q9I561	PA0884	Probable C4-dicarboxylate-binding periplasmic protein		0.01		
Q9I609	nirN	Probable c-type cytochrome		0.01		
Q9HZF5	PA3053	Probable hydrolytic enzyme		0.01		
Q9HVH0	PA4621	Probable oxidoreductase			0.01	
Q9I3T4	PA1410	Probable periplasmic spermidine/putrescine-binding protein		100	100	
Q9I6C2	PA0372	Probable zinc protease			0.062	
Q9I6J0	spuE	Spermidine-binding periplasmic protein SpuE		0.322		
P53641	sodB	Superoxide dismutase [Fe]		0.01		
G3XD55	pilY2	Type 4 fimbrial biogenesis protein PilY2			0.01	
G3XCZ3	pilZ	Type 4 fimbrial biogenesis protein PilZ		0.01		
G3XD59	PA4118	Uncharacterized protein		0.01	0.01	
Q9HT29	PA5545	Uncharacterized protein			100	
Q9HT41	PA5533	Uncharacterized protein			100	
Q9HU95	PA5088	Uncharacterized protein		0.138	0.01	
Q9HU96	PA5087	Uncharacterized protein		0.01	0.01	
Q9HUS9	PA4884	Uncharacterized protein			100	
Q9HUT9	PA4874	Uncharacterized protein		0.01		
Q9HVE9	PA4643	Uncharacterized protein			100	
Q9HVF2	PA4639	Uncharacterized protein			100	
Q9HVS6	PA4495	Uncharacterized protein		0.01		
Q9HWW2	PA4066	Uncharacterized protein		100	100	
Q9HXL0	PA3785	Uncharacterized protein			21.783	
Q9HYE2	PA3464	Uncharacterized protein			100	
Q9HYI4	PA3421	Uncharacterized protein			100	
Q9HYJ1	PA3414	Uncharacterized protein			100	
Q9HYJ2	PA3413	Uncharacterized protein			0.01	
Q9HYZ0	PA3250	Uncharacterized protein		0.01	0.01	
Q9HZ16	PA3224	Uncharacterized protein			100	
Q9HZ56	PA3178	Uncharacterized protein			0.01	
Q9HZD2	PA3076	Uncharacterized protein		0.01		
Q9I051	PA2792	Uncharacterized protein		0.01		
Q9I118	PA2464	Uncharacterized protein		4.523		
Q9I148	PA2434	Uncharacterized protein			100	
Q9I149	PA2433	Uncharacterized protein		0.371		
Q9I380	PA1645	Uncharacterized protein		0.01		
Q9I5B2	PA0827	Uncharacterized protein			100	
Q9I5H5	PA0754	Uncharacterized protein			100	
Q9I6C3	PA0371	Uncharacterized protein		100	100	
Q9I6E7	PA0346	Uncharacterized protein		100	100	
Q9I6N0	PA0259	Uncharacterized protein		100	100	
Q9I7A2	PA0028	Uncharacterized protein		0.01		
Q9I4L4	yfiR	YfiR			100	
Q9HXN9	PA3756	YkuD domain-containing protein		100	100	
Q9I5J7	PA0732	YkuD domain-containing protein		0.01		
Q9HT54	PA5519	HotDog ACOT-type domain-containing protein	Periplasmic/Cytoplasmic	0.01		
Q9HU45	PA5139	PBPb domain-containing protein		0.451		
Q9I6J4	spuA	Probable glutamine amidotransferase			100	
Q9I793	PA0039	Uncharacterized protein			5.972	
G3XD94	wbpA	UDP-N-acetyl-D-glucosamine 6-dehydrogenase	Membrane	100		
Q9HUE1	PA5028	AAA_31 domain-containing protein	CytoplasmicMembrane		0.01	
Q9I3F6	aer	Aerotaxis receptor Aer		100		pae02030 Bacterial chemotaxis
Q9I5M1	migA	Alpha-1,6-rhamnosyltransferase MigA			0.01	
Q9HT16	atpF	ATP synthase subunit b		100	100	
Q9I3N3	ccmE	Cytochrome c-type biogenesis protein CcmE		100	100	
Q9HYR4	PA3331	Cytochrome P450			0.01	
Q9X6V6	rlpA	Endolytic peptidoglycan transglycosylase RlpA		0.01	0.01	
Q51465	fliM	Flagellar motor switch protein FliM		0.01		pae02040 Flagellar assembly; pae02030 Bacterial chemotaxis
Q9HWI4	bfiS	Histidine kinase		0.01	0.01	
G3XD67	hcnA	Hydrogen cyanide synthase subunit HcnA		5.027		
Q9I345	PA1680	Hydrolase_4 domain-containing protein		0.01		
P45682	PA3623	Lipoprotein NlpD/LppB homolog			100	
Q9HZK8	nqrC	Na(+)-translocating NADH-quinone reductase subunit C			100	
Q9I0J9	nuoC	NADH-quinone oxidoreductase subunit C/D		100	100	
Q51546	pstB	Phosphate import ATP-binding protein PstB		0.01	0.01	
Q9I174	PA2407	Probable adhesion protein		0.01	7.105	
Q9I3C0	PA1601	Probable aldehyde dehydrogenase		0.01		
Q9HU32	PA5152	Probable ATP-binding component of ABC transporter		0.01	0.01	
Q9HZ51	PA3187	Probable ATP-binding component of ABC transporter		0.01	0.01	
Q9I031	PA2812	Probable ATP-binding component of ABC transporter		100		
Q51368	tonB	Protein TonB			100	
Q9I1N5	pslD	PslD		0.01	0.01	pae02025 Biofilm formation
Q9I0F1	PA2691	Pyr_redox_2 domain-containing protein		0.01		
G3XD25	mexC	Resistance-Nodulation-Cell Division (RND) multidrug efflux membrane fusion protein MexC		0.01		
P38107	mucA	Sigma factor AlgU negative regulatory protein		0.01		
Q9I3D4	sdhB	Succinate dehydrogenase (quinone)			100	
Q9HT45	PA5528	Tim44 domain-containing protein		4.963	0.01	
Q9I7B0	trkA	Trk system potassium uptake protein TrkA			0.01	
Q9HUE7	PA5022	Uncharacterized protein			100	
Q9HUK4	PA4961	Uncharacterized protein		0.01		
Q9HW29	PA4373	Uncharacterized protein			0.01	
Q9HXR3	PA3729	Uncharacterized protein		0.01	0.01	
Q9HZG8	PA3040	Uncharacterized protein		100		
Q9HZV2	PA2895	Uncharacterized protein		100	100	
Q9I3G7	PA1550	Uncharacterized protein		0.01		
Q9I413	PA1331	Uncharacterized protein		100		
Q9I5R2	PA0659	Uncharacterized protein			0.01	
Q9I6D5	PA0359	Uncharacterized protein			100	
Q9HVT1	PA4490	Uncharacterized protein PA4490			100	
Q9I1B3	PA2366	Uricase PuuD			0.01	pae02025 Biofilm formation
Q9HU14	cysQ	3'(2'),5'-bisphosphate nucleotidase CysQ	CytoplasmicMembrane/Cytoplasmic	0.01		
Q9I6V6	aer2	Aerotaxis transducer Aer2		0.01	0.01	pae02030 Bacterial chemotaxis

*Protein identifier and gene name refer to UniProtKB database; abundance ratio of the differentially expressed proteins in MDR and PDR strains compared to WT (100 = not identified in WT and 0.01 = identified only in WT); code and name of selected pathway retrieved by Kyoto Encyclopedia of Genes and Genomes database (KEGG, release 99.0, July 1, 2021, https://www.genome.jp/kegg/). Underlined proteins are discussed in the text*.

In order to gain insights into the processes related to these differentially expressed proteins, both literature data mining and functional analysis were performed highlighting 27 proteins, which were reported and discussed in the subsequent sections as representative of virulome (e.g., Tse2, Tse5, Tsi1, PilF, FliY, B-type flagellin, FliM, PyoS5), resistome (e.g., OprJ, LptD) and biofilm (e.g., AlgF, PlsD) pathways.

### Virulome Modulated Proteins

**Figure 5:**
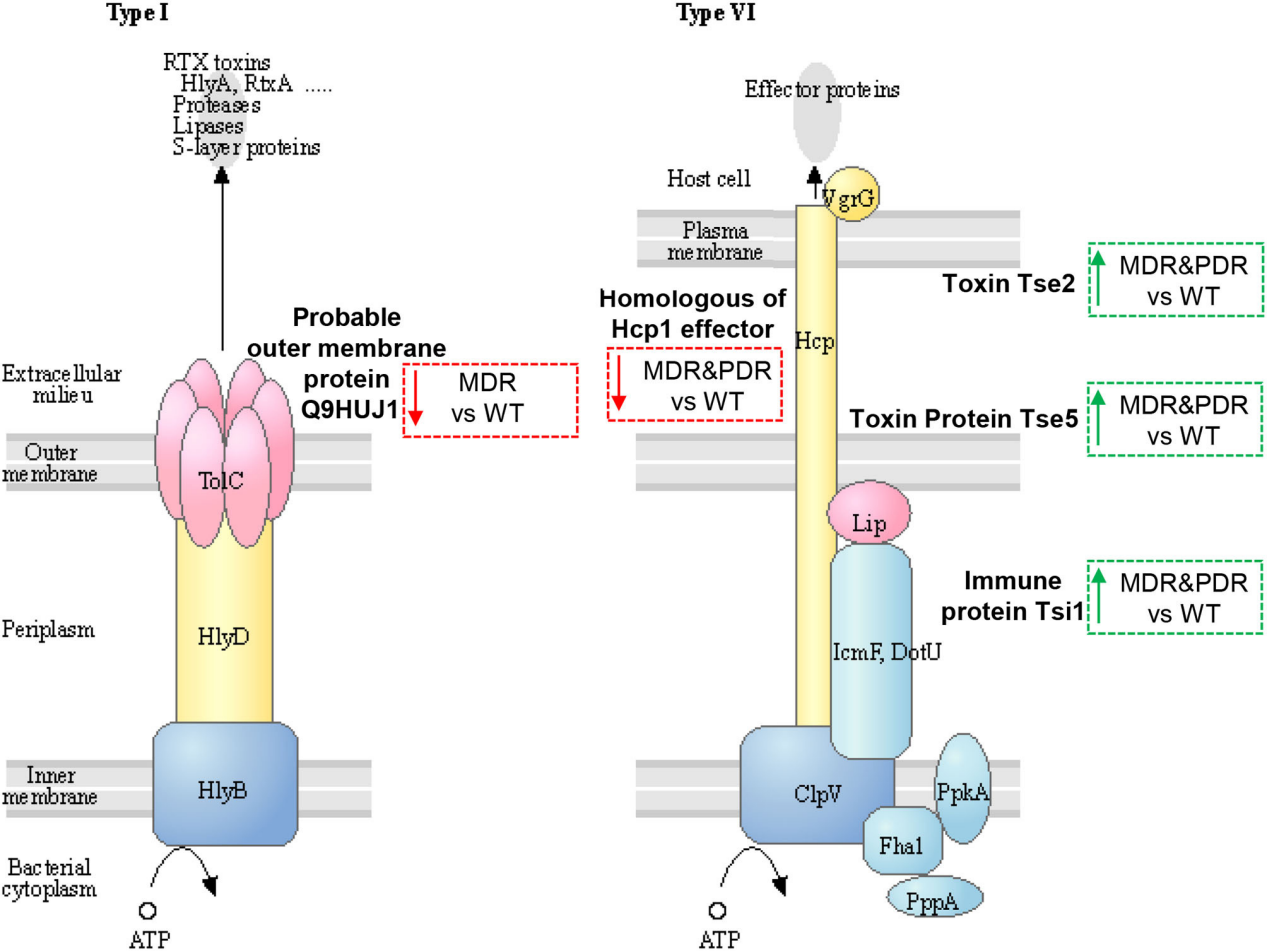
Sketch of two secretion systems (Type I and VI), transporting microbial substrates across membranes of Gram-negative bacteria in one step. Image modified from pae03070 KEGG (release 99.0, July 1, 2021) pathway depicting participating factors, proteins, complexes, and their subcellular distribution. MDR and PDR *P. aeruginosa* clinical strain identified proteins are evidenced by dashed box along with their shift of expression compared to WT strains (red dashed box and downward arrow = under-expression; green box and upward arrow = over-expression). Probable outer membrane protein (UniProtKB code Q9HUJ1, KEGG identifier PA4974) is localized in the outer membrane and involved in bacterial secretion system Type I. The identified Uncharacterized protein (Q9I1B2) is a homologous of Hcp1 family type VI secretion system effector and is mapped by KEGG software (KEGG identifier PA2367) as type VI secretion system secreted protein Hcp ortholog. Toxin Tse2 (Q9I0E0), Toxin protein Tse5 (Q9I0F4) and Immune protein Tsi1 (Q9I2Q0) are other members of the type VI secretion system.

Three identified proteins were associated to the secretion system, namely Toxin Tse2, Toxin protein Tse5 and Immune protein Tsi1, (Q9I0E0, Q9I0F4, and Q9I2Q0), over-expressed in both comparisons MDR and PDR vs. WT and belonging to T6SS (47, 48). Another two proteins were part of the T1SS: the homologous of the Hcp1 family type VI secretion system effector (Q9I1B2), under-expressed in both comparisons, and the Probable outer membrane protein (Q9HUJ1), under-expressed only in MDR vs. WT (Figure 5).

Tse2, actively interacting with Hcp1 (49), is a toxic molecule directed against other prokaryotes by inhibiting their growth (50, 51). Tse5 interacts with the VgrG (Valine-glycine repeat G) (52, 53) and shows a direct toxicity against bacteria (54). Tsi1 binds Tse1 by active site occlusion and inhibits enzyme activity, overcoming the destructive action of the Tse1 (52, 53, 55). In fact, *P. aeruginosa* generates periplasmic immune proteins called Tsi(n) which neutralizes Tse(n) toxicity exerted by sister bacteria. In particular, Tsi1, identified in our study, is a cysteine peptidase, structurally related to the N1pC/P60 hydrolase superfamily, which is involved in peptidoglycan degradation. The secreted Hcp1 could be used as a marker of a functional T6SS because of previous evidences (56). Indeed, Hcp1 was found in CF sputum and also antibodies against Hcp1 were detected in the serum of the same patients (11).

The enzyme Uricase PuuD (Q9I1B3), under-expressed in PDR vs. WT, converts the uric acid to allantoin, and allows PA survival in the CF lung which is rich in purines as nitrogen source (57, 58). Moreover, the Uricase, dropping the uric acid, which is an alarmin triggering chemotaxis and NLRP3 inflammasome (59, 60), may promote microbial escape from host response. Therefore, the Uricase might have a double function: metabolic, to ensure self-survival; and acting as a virulence factor explaining its over-expression in WT strains.

Relating to mobility and adhesion processes, the Type IV pilus (T4P) assembly protein PilF (Q9HXJ2) resulted in over-expression in WT. This outer membrane protein is essential for the biogenesis of the T4P promoting the insertion of PilQ in the outer membrane as a pivotal host colonization step (61). Indeed, the T4P complex has a crucial function in WT strains for host invasion and settlement as observed for less infectious mutants with impaired T4P (62). Therefore, the pilus appeared under-expressed in the MDR and PDR groups, as expected for long-term colonization PA strains (13).

Regarding the flagellar assembly system, the L-cysteine transporter of ABC system FliY (Q9I6H7) was under-expressed in PDR vs. WT; the B-type flagellin (P72151) and Flagellar motor switch protein FliM (Q51465) were under-expressed in MDR vs. WT (Supplementary Figure 1A). Flagella structure is made by a membrane complex, a flexible-hook, and a flagellin filament (63). Flagellin, serotypes a and b, triggers inflammation through Toll-like receptor 5 and it has been studied as a vaccine target (64). FliM interplays with the chemosensory framework for switching (63). The switch complex of the flagellar C ring, containing FliM, comprises also the protein FliY, a member of the phosphatase family, essential to the flagellar switching (65–67). Flagella are important virulence factors for PA and flagellum-negative strains are less virulent than flagellum-positive strains in a burned-mouse model (68). However, they are not advantageous in strains triggering chronic infection.

Involved in the chemiotaxis process with FliM, we identified the Aerotaxis transducer Aer2 (Q9I6V6) as under-expressed in MDR and PDR vs. WT comparisons. Other components of the chemiotaxis process were differentially expressed: the Probable binding protein component of ABC transporter (Q9HVS5) was under-expressed in MDR vs. WT; the Aerotaxis receptor Aer (Q9I3F6) was over-expressed in MDR vs. WT; and the Binding protein component of ABC ribose transporter (Q9I2F8) was over-expressed in PDR vs. WT (Supplementary Figure 1B). Aer in PA is a family of receptors able to sense cellular energy. Its transducer Aer2, a soluble receptor, is reported to be crucial for host infection although its role is still unclear (69).

Among other virulence factors we identified Pyocin S5 (Q9I4Y4, PyoS5) over-expressed in MDR and PDR vs. WT, Fe(3+)-pyochelin receptor (P42512, FptA) and Protein TonB (Q51368, TonB) both over-expressed in PDR vs. WT. In the competition for the ecological niche, bacteria develop multiple mechanisms to ensure their survival and predominance. PA produces siderophores, such as pyoverdine, pyochelin and pyocianin (i.e., PyoS5), to bind iron and to transport it from the extracellular region into the bacterium through the outer membrane receptor FptA, a TonB-dependent transporter (70–76).

These results highlight PA adaptation processes through different phases, from onset to chronic infection stages. In fact, during early infection the bacterium needs pili and flagella to move itself, settle and colonize the CF lung. However, these bacterial structures trigger the host immune response leading to the activation of the pro-inflammatory MyD88 pathway and neutrophil recruitment through the Toll-like receptor 5 (TLR5) binding. Moreover, translocation of the flagellin into host cells by the T3SS also activate the NLRC4-inflammasome (77). Despite flagellar structures being essential during initial infection, their proteins becomes disadvantageous for the bacterium in the later stages of infection for both their uselessness in the infectious path and their ability to activate the host's immune response, to be avoided. Thus, PA becomes effective in the evasion process, favoring loss of flagellar protein recognition by the host, hence losing pili and flagella during chronic infection. At the same time the bacterium advances toward chronicity, launching alternative virulence strategies (i.e., toxins and pyocianin).

### Resistome Modulated Proteins

**Figure 6:**
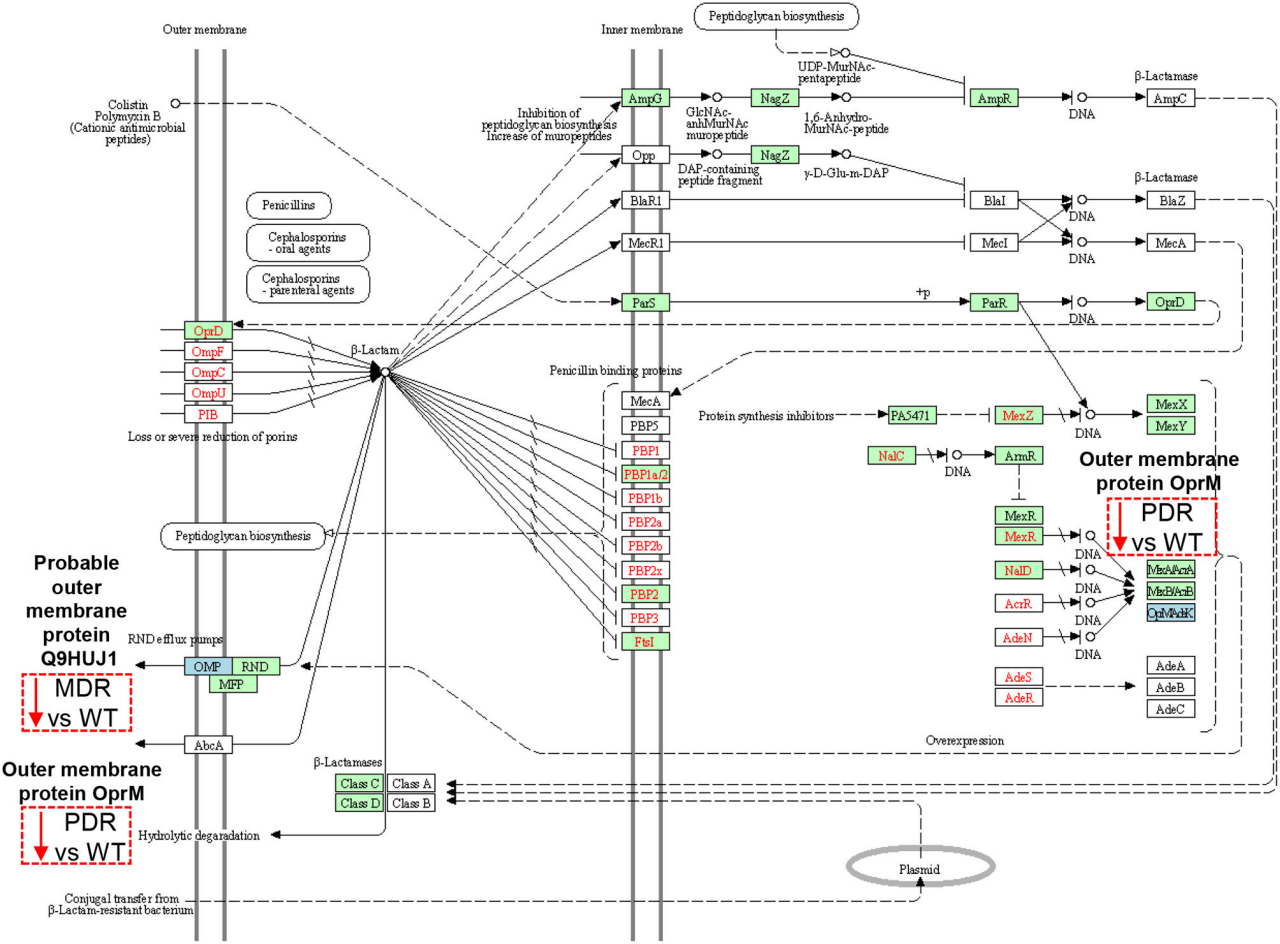
Sketch of the β-Lactam resistance pathway. Image modified from pae01501 KEGG (release 99.0) pathway showing involved factors/effectors, their subcellular distribution and their molecular interactions, reactions and relation networks. MDR and PDR *P. aeruginosa* clinical strain identified proteins are represented by a dashed box along with their shift of expression compared to WT strains (red dashed box and downward arrow = under-expression). Among the outer membrane proteins, we identified a Probable outer membrane protein (UniProtKB code Q9HUJ1, KEGG identifier PA4974) and the Outer membrane protein OprM (Q51487, PA0427), both involved in the resistance-nodulation division (RND) efflux pump mechanisms at their outer membrane subcellular localization. OprM modulation may also result from intrinsic properties of organisms, through gene mutations, and through plasmid- and transposon-specified genes at the DNA level (OpMAdeK system). Light blue box = input protein, differentially expressed in MDR or PDR vs. WT. Green box = *P. aeruginosa*-specific pathway. Red characters = resistance associated gene variants.

The Probable outer membrane protein (Q9HUJ1), under-expressed in MDR vs. WT, https://ops.hindawi.com/author/1744408/ is also involved in the β-Lactam resistance pathway and mapped within the resistance-nodulation division (RND) efflux pumps together with the Outer membrane protein OprM (Q51487), under-expressed in PDR vs. WT (Figure 6). In the Transporter Classification Database (TCDB) the Q9HUJ1 protein is identified as the OpmH protein involved in triclosan resistance efflux pump TriABC-OpmH, a triclosan-specific pump requiring two membrane fusion proteins for function and whose expression is caused by a promoter-up mutation (78). Triclosan, a chemical with antibacterial properties used in the past as an addition ingredient in soaps, toothpaste and cosmetics, has been recently banned from the market and AR of our MDR strains is not associated to the expression of the Q9HUJ1 protein.

Several mechanisms, instead, determine AR in PA, including those related to efflux pumps (79). Their over-expression is associated with multi-drug resistance and modulation of bacterial function such as quorum sensing and motility (80–82).

OprM pumps out several classes of antibiotics such as quinolones, tetracyclines, and chloramphenicol (80, 83, 84). Despite the expectation of an increase in its expression in antibiotic resistant strains (85), our data show the opposite trend. OprM is also the receptor for bacteriophages. To protect itself, PA is forced to down-regulate several phage receptors, OprM included (86), as happen in the CF lung milieu in which a large population of bacteriophages is naturally occurring (87, 88). Moreover, OprM may promote the selection of mutations in *oprD*, whose reduced expression is known to cause carbapenem resistance in PA (89). Thus, the observation of the decreased expression of such protein in the PDR group may also be associated to carbapenem resistance.

OprJ (Q51397), constituting the MexCD-OprJ system, showed over-expressed in both MDR and PDR vs. WT comparisons. This efflux pump is involved in the outflow of antibiotics, such as fluoroquinolone, macrolides, cefepime, and tetracyclines (90, 91). Indeed, its over-expression in our antibiotic resistant groups, both MDR and PDR, is consistent with the ciprofloxacin, levofloxacin and cefepime resistance we observed in our strains. Moreover, its over-expression correlates to a decrement of virulence factors involved in quorum sensing (79, 80, 83, 84). Both, OprJ over-expression and virulence factors' reduction can be consistent with chronic colonization of MDR and PDR PA.

Gram-negative bacteria display integral outer membrane proteins (OMPs) that need the Omp85, family characterized by POTRA (polypeptide-transport-associated) tandem motifs, for proper folding and insertion (92, 93). The OM is essential in Gram-negative bacteria as barrier protection and contributes to the establishment of AR vigorously blocking antibiotic passage (94). The POTRA domain-containing proteins, such as Q9HVG7, over-expressed in MDR and PDR strains compared to WT, and Q9I119, over-expressed in MDR vs. WT strains, may reflect this condition (95).

Another protein involved in OM robustness, the LPS-assembly protein LptD (Q9I5U2), a β-barrel protein, showed over-expressed in MDR vs. WT. This protein is involved in the transport of the LPS to the outer layer of the membrane, regulating its permeability and conferring antibiotic resistance (96, 97). In fact, permeability of bacterial membranes is crucial for antibiotic susceptibility, whereas higher permeability is associated with antibiotic susceptibility and lower permeability with greater resistance. Thus, LptD protein over-expression in our MDR group may be one of the factors that could increase the antibiotic resistance in our strains.

### Biofilm Modulated Proteins

PA strains were probed for their ability to produce biofilm, classifying them as “strong,” “moderate,” and “weak” biofilm producers. Seventy percent of WT, 66.7% of MDR and 42.9% of PDR groups were classified as “strong” biofilm producers (Table 3). Notably, 57.1% of the PDR clinical isolates were “weak” biofilm producers. Moreover, considering the two AR groups together, 56.3% were classified as “strong,” 18.8% as “moderate” and 25.0% as “weak” biofilm producers. The biofilm assay corroborated the under-expression of biofilm-associated proteins highlighted by the proteomics approach in MDR and PDR strains.

**Table 3:** Classification of *P. aeruginosa* strains based on their biofilm formation ability.

**Group**	**Strain**	**Biofilm producer**	**OD**	
			**Mean**	**Standard deviation**
MDR	MDR_01	Strong	0.6553	0.1220
	MDR_02	Moderate	0.3549	0.0663
	MDR_03	Strong	1.7403	0.2467
	MDR_04	Strong	0.8143	0.1042
	MDR_05	Strong	0.7144	0.1203
	MDR_06	Moderate	0.3242	0.0987
	MDR_07	Strong	0.6074	0.2202
	MDR_08	Strong	0.5749	0.0720
	MDR_09	Strong	0.9628	0.2274
PDR	PDR_01	Weak	0.1424	0.0152
	PDR_02	Strong	1.0849	0.1210
	PDR_03	Weak	0.1483	0.0269
	PDR_04	Weak	0.1418	0.0178
	PDR_05	Strong	1.0316	0.1991
	PDR_06	Weak	0.1387	0.0234
	PDR_07	Moderate	0.2436	0.0651
WT	WT_01	Moderate	0.2632	0.0297
	WT_02	Strong	0.6227	0.1362
	WT_03	Strong	1.0899	0.1600
	WT_04	Strong	0.6154	0.0589
	WT_05	Strong	0.6077	0.0813
	WT_06	Strong	0.5134	0.0876
	WT_07	Moderate	0.3538	0.0891
	WT_08	Strong	0.9716	0.1035
	WT_09	Strong	0.9452	0.1728
	WT_10	Moderate	0.2310	0.0339

*Optical Density (OD) values, after crystal violet staining, were recorded at 590 nm*.

The Hcp1 homolog (Q9I1B2), besides the secretion system, was indeed mapped into the biofilm formation pattern with PslD (Q9I1N5), under-expressed in MDR vs. WT and PDR vs. WT, and with Uricase PuuD (Q9I1B3), under-expressed in PDR vs. WT (Supplementary Figure 1C).

Also Alginate biosynthesis protein AlgF (Q06062) and the Alginate_lyase2 domain-containing protein (Q9I2V8) were under-expressed in MDR PDR strains. On the contrary, the periplasmic protein Alginate lyase (Q06749) was over-expressed in PDR vs. WT.

Results of the biofilm assay, demostrating WT strains as “stronger” biofilm producers, suggest there is some role for the biofilm in the context of early infection. As previously discussed, there are dynamic interactions between PA structures, such as flagella, and the host leading to an activation of the host immune response. Biofilms establish an organized community of bacteria encapsulated in extracellular polymeric substance (EPS) matrices protecting them from hostile and unstable environmental conditions, including host defense (98). Hence, bacterial proteins involved in initiating the host immune response, such as those of the flagella, may be enclosed within the biofilm matrix and hidden to host recognition (77). Moreover, biofilm is able to immobilize neutrophils providing PA with protection from phagocytosis (98). Thus, biofilms may protect WT PA from host immune response and hypothetical antimicrobial molecules, and advantageously promote its survival, immunological escape and ultimately the propagation of the infection.

## Discussion

Analysis of the surface proteins may provide new findings for comprehension of bacterial fitness, bacteria-host and bacteria-environment interactions. For this reason we pursued a shaving proteomic approach that allowed the characterization of PA surface proteins and related functional profiles of CF clinical isolates with different antimicrobial susceptibility patterns and lung colonization phases.

During CF airways chronic infection, PA adapts its phenotype within the distinct anatomical niches of the lung. This “adaptive radiation” is a definite event (10) under which the bacterium, endowed with high plasticity, puts in place several mechanisms to acquire the capability to survive in an ecological niche characterized by low oxygen, nutrients limitation, osmotic and oxidative stress, competition from other colonizers and other adversity. It is commonly accepted that the phenomenon includes early and late modifications (13). In the course of early infection the presence of flagella and pili allows the bacterium to reach and settle in the pulmonary niche; later, PA reduces its early settlement factors in order to modulate bacterial fitness activating a more complex machinery able to guarantee its survival in the CF lung (99). Virulence factors, such as toxins and pyocianins, modifications of the membrane assembly and robustness, changes in the biofilm lifestyle, and antibiotic susceptibility reduction, are chronic pathophenotype traits. Re-arrangements of the proteins on the surface of the bacterium reflect adaptation mechanisms, suggesting that chronic antibiotic resistance of PA isolates may express a different surface protein layout compared to early antibiotic sensitive PA strains.

Our results highlight that MDR and PDR chronic PA strains were deprived of several early virulence factors such as pili and flagella, while expressing later virulence factors such those belonging to bacterial secretion systems. On the contrary, we observed an unexpected low number of resistome-related proteins in the chronic antibiotic resistant strains with four out of six over-expressed proteins. However, we should recall AR is a notably complicated process in PA and the resistance to each class of antibiotics may be mediated by multiple mechanisms which include efflux pumps, genes mutations, plasmids, β-lactamases, OprD alterations and permeability modifications of the bacterial membranes; several types of resistance may be present in a strain at the same time mediating the same antibiotic class resistance (10). In our MDR and PDR strains, the most significant over-expressed protein was OprJ (Q51397), a component of the efflux pump MexCD-OprJ system. The resistance of the investigated strains to fluoroquinolones and cefepime may be consistent with the over-expression of this pump. Indeed, an increase of drug output through modification of this efflux pump activity is the primary mechanism conferring ciprofloxacin and levofloxacin resistance in PA (91). On the other hand, over-expression of the LPS transport protein LptD in MDR group and of the two Q9HVG7 and Q9I119 POTRA domain-containing proteins in MDR and PDR and in MDR vs. WT strains, respectively, is involved in the permeability modification of the bacterial membranes, thus conferring antibiotic resistance to several classes of antimicrobial, including β-lactams. Notably, we identified two antibiotic-resistance associated proteins (Outer membrane protein Q9HUJ1 and Q51487) under-expressed in our MDR and PDR isolates, respectively. Previously studies on PA longitudinal strains demonstrated that a fully adapted phenotype is observed after 20 years of colonization. Since the chronic strains included in our study actually belong to the first two decades after PA infection (<15 years), we can infer that protein expression associated to antibiotic resistance is not yet stabilized (100).

Results of the biofilm assay demonstrated that MDR and PDR strains are “weaker” biofilm producers supporting the proteomic data of under-expression of biofilm-associated proteins (Hcp1 family type VI secretion system effector protein, PslD, Uricase PuuD, Alginate biosynthesis protein AlgF and Alginate_lyase2 domain-containing protein).

The under-expression of the Hcp1 in MDR and PDR groups is consistent with literature since T6SS has been described as involved also in biofilm formation (101) and the deletion of the *hcp1* gene was associated with defective strains in biofilm production (102). Moreover, the under-expression of PslD might suggest that our MDR and PDR non-mucoid clinical strains may produce a biofilm composed by other exopolysaccharides than alginate. In fact, *psl* gene cluster is involved in biofilm formation during the early infection phase (103–110). Only during later stages of infection, do the algACD operon take control of the biofilm formation starting the production of alginate (110), a linear copolymer of mannuronic and guluronic acid, which is the key exopolysaccharide of PA biofilm produced by mucoid strains from long term colonization CF patients (111).

Indeed, PA mucoid strains are the expression of the evolution of the bacterium to adaptating in the CF lung where the alginate is involved in bacterial defense against both the dangerous environment and antibiotic molecules, promoting bacterial long-term colonization. In our antibiotic-resistant non-mucoid strains, under-expression of such proteins remarks the fact that the adaptation process of the bacterium to the CF lung environment is always ongoing and that the mucoid phenotype is not yet achieved. However, we noticed that the periplasmic protein Alginate lyase (AlgL) was over-expressed in the PDR strains. We speculate that since PA is constantly evolving, the AlgL over-expression may be part of the initial process toward acquiring a later mucoid phenotype, a transition relying also on severity of lung disease and anti-pseudomonal antibiotics use (112).

In conclusion, the surfaceome analyses of our isolates, rather than expressing a protein profile associated to antibiotic resistance, is a mirror of the processes of bacterium persistence into the peculiar environment of the CF lung. Indeed, PA is a complex and dynamic bacterium and it is extremely hard to find its characteristic traits, especially associated to antibiotic resistance.

To the best of our knowledge, this is the first study using shaving proteomics to characterize a large set of PA clinical isolates. Our evidences suggest that their adaptation processes may modulate the surface protein expression profile in Cystic Fibrosis in response to different lung stressors.

## Data Availability Statement

The data presented in the study are deposited in the ProteomeXchange repository, accession number PXD030040.

## Ethics Statement

This study was approved by the Ethical Committee of Bambino Gesù Children's Hospital, Rome, Italy; protocol code 202105_INNOV_ONETTI.

## Author Contributions

LP and EF: conceptualization and supervision. AO, LP, and EF: funding acquisition. AM, VM, NE, SL, MR, SG, LS, and GV: investigation. VM and SG: visualization. AM and VM: writing—original draft. AM, VM, AO, LP, and EF: writing—review and editing. All authors contributed to the article and approved the submitted version.

## Funding

This research was funded by OFFICIUM onlus Lega Italiana Fibrosi Cistica Lazio to EF, Ricerca Corrente of Minister of Italian Health grant code RC2020_ICG_ONETTI and RC202105_INNOV_ONETTI to AO, and RC2021_MODALE_PUTIGNANI to LP.

## Conflict of Interest

SG was employed by GenomeUp. The remaining authors declare that the research was conducted in the absence of any commercial or financial relationships that could be construed as a potential conflict of interest.

## Publisher's Note

All claims expressed in this article are solely those of the authors and do not necessarily represent those of their affiliated organizations, or those of the publisher, the editors and the reviewers. Any product that may be evaluated in this article, or claim that may be made by its manufacturer, is not guaranteed or endorsed by the publisher.

## Supplementary Material

Supplementary Figure 1 Image of biofilm formation **(A)**, flagellar assembly **(B)** and bacterial chemotaxis **(C)** KEGG (release 99.0) pathway. Light blue box = input protein, differentially expressed in MDR or PDR vs. WT. HSI-I = KEGG identifier PA2366, UniProtKB code Q9I1B3, Uricase PuuD, and PA2367, Q9I1B2, Hcp1 homologous Uncharacterized protein; Psl = PA2234, Q9I1N5, PslD; FliM = PA1443, Q51465, Flagellar motor switch protein FliM; FliC = PA1092, P72151, B-type flagellin; FliY = PA0314, Q9I6H7, L-cysteine transporter of ABC system FliY; MCP = PA0176, Q9I6V6, Aerotaxis transducer Aer2; Aer = PA1561, Q9I3F6, Aerotaxis receptor Aer; RbsB = PA1946, Q9I2F8, Binding protein component of ABC ribose transporter; DppA = PA4496, Q9HVS5, Probable binding protein component of ABC transporter. Green box = *P. aeruginosa*-specific pathway.

Supplementary Table 1 List of patients' features and *P. aeruginosa* isolated strains.

Supplementary Table 2 List of identified proteins by nLC-ESI-MS/MS.

Supplementary Table 3 List of published shaving proteomics-based study related to Gram-positive and Gram-negative bacteria. Strains, shaving conditions and information about the identified proteins are listed.

Supplementary Table 4 Data matrix of LFQ protein abundance intensities.

Supplementary Data Sheet 1 List of identified proteins differentially expressed and their distribution in the two comparisons (MDR vs. WT and PDR vs. WT), highlighting shared and specific proteins, and their subcellular localization.
