# Supplementary figures and images for: A Shaving Proteomic Approach to Unveil Surface Proteins Modulation of Multi-Drug Resistant Pseudomonas aeruginosa Strains Isolated From Cystic Fibrosis Patients
Source: Front Med (Lausanne). 2022 Mar 9;9:818669. doi: 10.3389/fmed.2022.818669 (PMC8959810; doi:10.3389/fmed.2022.818669)

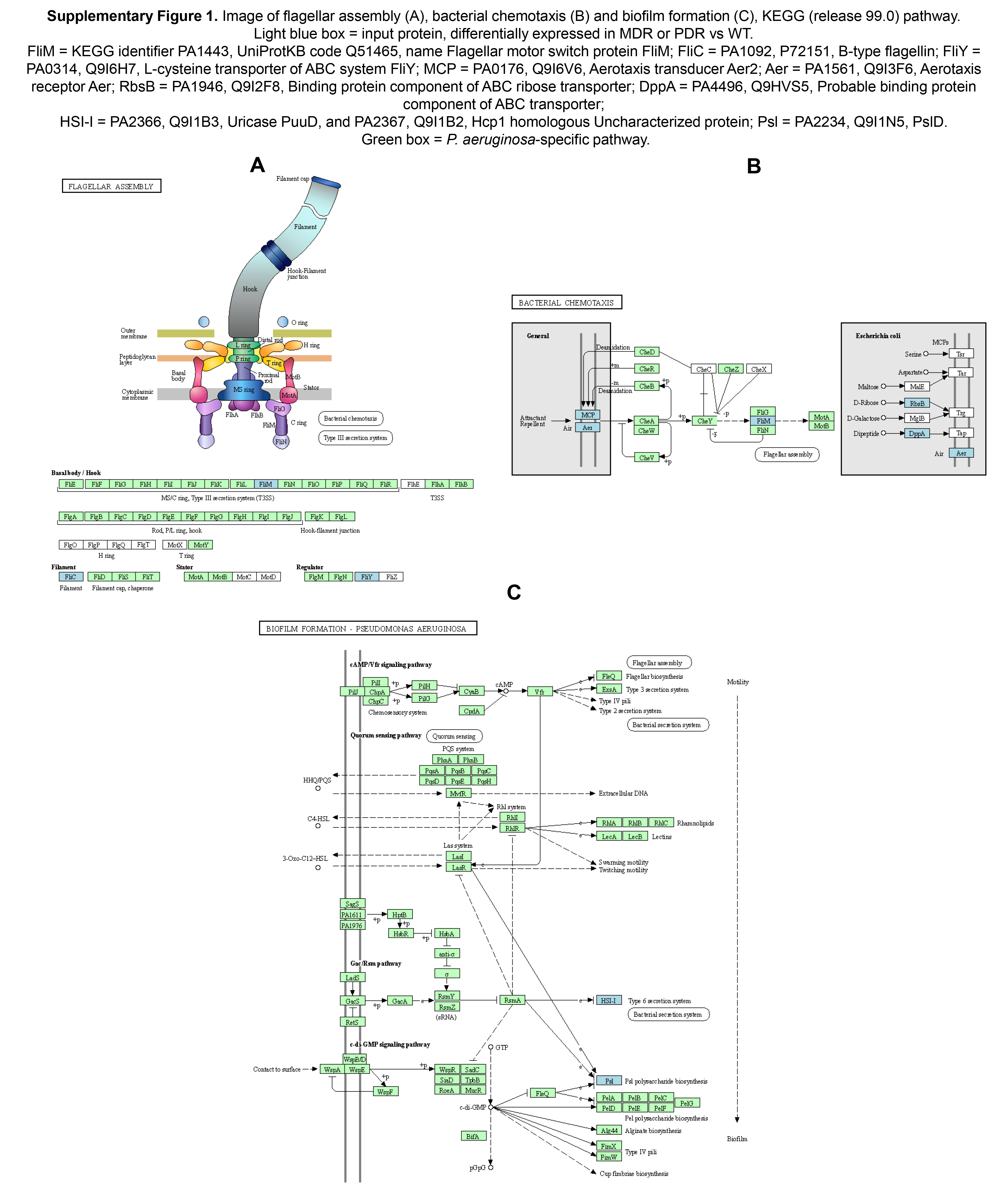

Supplement: Supplementary Figure 1 — Image of biofilm formation (A), flagellar assembly (B) and bacterial chemotaxis (C) KEGG (release 99.0) pathway. Light blue box = input protein, differentially expressed in MDR or PDR vs. WT. HSI-I = KEGG identifier PA2366, UniProtKB code Q9I1B3, Uricase PuuD, and PA2367, Q9I1B2, Hcp1 homologous Uncharacterized protein; Psl = PA2234, Q9I1N5, PslD; FliM = PA1443, Q51465, Flagellar motor switch protein FliM; FliC = PA1092, P72151, B-type flagellin; FliY = PA0314, Q9I6H7, L-cysteine transporter of ABC system FliY; MCP = PA0176, Q9I6V6, Aerotaxis transducer Aer2; Aer = PA1561, Q9I3F6, Aerotaxis receptor Aer; RbsB = PA1946, Q9I2F8, Binding protein component of ABC ribose transporter; DppA = PA4496, Q9HVS5, Probable binding protein component of ABC transporter. Green box = P. aeruginosa-specific pathway. [file Image_1.TIF]
